# Comparison of age-specific cataract prevalence in two population-based surveys 6 years apart

**DOI:** 10.1186/1471-2415-6-17

**Published:** 2006-04-20

**Authors:** Ava Grace Tan, Jie Jin Wang, Elena Rochtchina, Paul Mitchell

**Affiliations:** 1 Centre for Vision Research, Westmead Millennium Institute, Department of Ophthalmology, University of Sydney, Westmead Hospital, Westmead, NSW, Australia

## Abstract

**Background:**

In this study, we aimed to compare age-specific cortical, nuclear and posterior subcapsular (PSC) cataract prevalence in two surveys 6 years apart.

**Methods:**

The Blue Mountains Eye Study examined 3654 participants (82.4% of those eligible) in cross-section I (1992–4) and 3509 participants (75.1% of survivors and 85.2% of newly eligible) in cross-section II (1997–2000, 66.5% overlap with cross-section I). Cataract was assessed from lens photographs following the Wisconsin Cataract Grading System. Cortical cataract was defined if cortical opacity comprised ≥ 5% of lens area. Nuclear cataract was defined if nuclear opacity ≥ Wisconsin standard 4. PSC was defined if any present. Any cataract was defined to include persons who had previous cataract surgery. Weighted kappa for inter-grader reliability was 0.82, 0.55 and 0.82 for cortical, nuclear and PSC cataract, respectively. We assessed age-specific prevalence using an interval of 5 years, so that participants within each age group were independent between the two surveys.

**Results:**

Age and gender distributions were similar between the two populations. The age-specific prevalence of cortical (23.8% in 1^st^, 23.7% in 2^nd^) and PSC cataract (6.3%, 6.0%) was similar. The prevalence of nuclear cataract increased slightly from 18.7% to 23.9%. After age standardization, the similar prevalence of cortical (23.8%, 23.5%) and PSC cataract (6.3%, 5.9%), and the increased prevalence of nuclear cataract (18.7%, 24.2%) remained.

**Conclusion:**

In two surveys of two population-based samples with similar age and gender distributions, we found a relatively stable cortical and PSC cataract prevalence over a 6-year period. The increased prevalence of nuclear cataract deserves further study.

## Background

Age-related cataract is the leading cause of reversible visual impairment in older persons [1-6]. In Australia, it is estimated that by the year 2021, the number of people affected by cataract will increase by 63%, due to population aging [7]. Surgical intervention is an effective treatment for cataract and normal vision (> 20/40) can usually be restored with intraocular lens (IOL) implantation.

Cataract surgery with IOL implantation is currently the most commonly performed, and is, arguably, the most cost effective surgical procedure worldwide. Performance of this surgical procedure has been continuously increasing in the last two decades. Data from the Australian Health Insurance Commission has shown a steady increase in Medicare claims for cataract surgery [8]. A 2.6-fold increase in the total number of cataract procedures from 1985 to 1994 has been documented in Australia [9]. The rate of cataract surgery per thousand persons aged 65 years or older has doubled in the last 20 years [8,9]. In the Blue Mountains Eye Study population, we observed a one-third increase in cataract surgery prevalence over a mean 6-year interval, from 6% to nearly 8% in two cross-sectional population-based samples with a similar age range [10]. Further increases in cataract surgery performance would be expected as a result of improved surgical skills and technique, together with extending cataract surgical benefits to a greater number of older people and an increased number of persons with surgery performed on both eyes.

Both the prevalence and incidence of age-related cataract link directly to the demand for, and the outcome of, cataract surgery and eye health care provision. This report aimed to assess temporal changes in the prevalence of cortical and nuclear cataract and posterior subcapsular cataract (PSC) in two cross-sectional population-based surveys 6 years apart.

## Methods

The Blue Mountains Eye Study (BMES) is a population-based cohort study of common eye diseases and other health outcomes. The study involved eligible permanent residents aged 49 years and older, living in two postcode areas in the Blue Mountains, west of Sydney, Australia. Participants were identified through a census and were invited to participate. The study was approved at each stage of the data collection by the Human Ethics Committees of the University of Sydney and the Western Sydney Area Health Service and adhered to the recommendations of the Declaration of Helsinki. Written informed consent was obtained from each participant.

Details of the methods used in this study have been described previously [11]. The baseline examinations (BMES cross-section I) were conducted during 1992–1994 and included 3654 (82.4%) of 4433 eligible residents. Follow-up examinations (BMES IIA) were conducted during 1997–1999, with 2335 (75.0% of BMES cross section I survivors) participating. A repeat census of the same area was performed in 1999 and identified 1378 newly eligible residents who moved into the area or the eligible age group. During 1999–2000, 1174 (85.2%) of this group participated in an extension study (BMES IIB). BMES cross-section II thus includes BMES IIA (66.5%) and BMES IIB (33.5%) participants (n = 3509).

Similar procedures were used for all stages of data collection at both surveys. A questionnaire was administered including demographic, family and medical history. A detailed eye examination included subjective refraction, slit-lamp (Topcon SL-7e camera, Topcon Optical Co, Tokyo, Japan) and retroillumination (Neitz CT-R camera, Neitz Instrument Co, Tokyo, Japan) photography of the lens. Grading of lens photographs in the BMES has been previously described [12]. Briefly, masked grading was performed on the lens photographs using the Wisconsin Cataract Grading System [13]. Cortical cataract and PSC were assessed from the retroillumination photographs by estimating the percentage of the circular grid involved. Cortical cataract was defined when cortical opacity involved at least 5% of the total lens area. PSC was defined when opacity comprised at least 1% of the total lens area. Slit-lamp photographs were used to assess nuclear cataract using the Wisconsin standard set of four lens photographs [13]. Nuclear cataract was defined when nuclear opacity was at least as great as the standard 4 photograph. Any cataract was defined to include persons who had previous cataract surgery as well as those with any of three cataract types. Inter-grader reliability was high, with weighted kappa 0.82 for cortical cataract, 0.55 (simple kappa 0.75) for nuclear cataract and 0.82 for PSC grading. The intra-grader reliability for nuclear cataract was assessed with simple kappa 0.83 for the senior grader who graded nuclear cataract at both surveys. All PSC cases were confirmed by an ophthalmologist (PM).

In cross-section I, 219 persons (6.0%) had missing or ungradable Neitz photographs, leaving 3435 with photographs available for cortical cataract and PSC assessment, while 1153 (31.6%) had randomly missing or ungradable Topcon photographs due to a camera malfunction, leaving 2501 with photographs available for nuclear cataract assessment. Comparison of characteristics between participants with and without Neitz or Topcon photographs in cross-section I showed no statistically significant differences between the two groups, as reported previously [12]. In cross-section II, 441 persons (12.5%) had missing or ungradable Neitz photographs, leaving 3068 for cortical cataract and PSC assessment, and 648 (18.5%) had missing or ungradable Topcon photographs, leaving 2860 for nuclear cataract assessment.

Data analysis was performed using the Statistical Analysis System (SAS, SAS Institute, Cary, NC, USA). Age-adjusted prevalence was calculated using direct standardization of the cross-section II population to the cross-section I population. We assessed age-specific prevalence using an interval of 5 years, so that participants within each age group were independent between the two cross-sectional surveys.

## Results

Characteristics of the two survey populations have been previously compared [14] and showed that age and sex distributions were similar. Table 1 compares participant characteristics between the two cross-sections. Cross-section II participants generally had higher rates of diabetes, hypertension, myopia and more users of inhaled steroids.

**Table 1:** Participant characteristics.

Characteristics	Cross-section I		Cross-section II	
	
	n	%	n	%
Age (mean)	(66.2)		(66.7)	
50–54	485	13.3	350	10.0
55–59	534	14.6	580	16.5
60–64	638	17.5	600	17.1
65–69	671	18.4	639	18.2
70–74	538	14.7	572	16.3
75–79	422	11.6	407	11.6
80–84	230	6.3	226	6.4
85–89	100	2.7	110	3.1
90+	36	1.0	24	0.7
				
Female	2072	56.7	1998	57.0
				
Ever Smokers	1784	51.2	1789	51.2
				
Use of inhaled steroids	370	10.94	478	13.8^
				
History of:				
Diabetes	284	7.8	347	9.9^
Hypertension	1669	46.0	1825	52.2^
				
Emmetropia*	1558	42.9	1478	42.2
Myopia*	442	12.2	495	14.1^
Hyperopia*	1633	45.0	1532	43.7

n = number of persons affected

* best spherical equivalent refraction correction

^ P < 0.01

**Figure 1:**
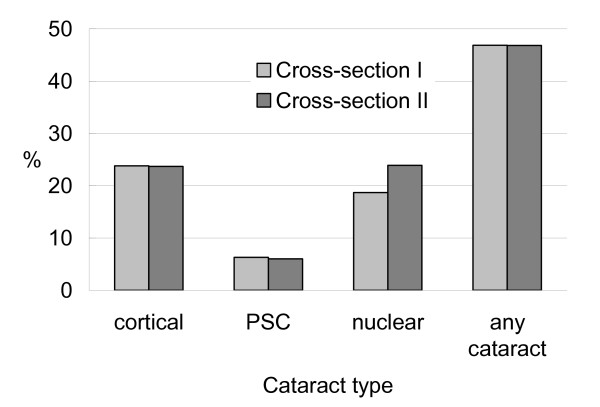
Cataract prevalence in cross-sections I and II of the Blue Mountains Eye Study.

Cataract prevalence rates in cross-sections I and II are shown in Figure 1. The overall prevalence of cortical cataract was 23.8% and 23.7% in cross-sections I and II, respectively (age-sex adjusted P = 0.81). Corresponding prevalence of PSC was 6.3% and 6.0% for the two cross-sections (age-sex adjusted P = 0.60). There was an increased prevalence of nuclear cataract, from 18.7% in cross-section I to 23.9% in cross-section II over the 6-year period (age-sex adjusted P < 0.001). Prevalence of any cataract (including persons who had cataract surgery), however, was relatively stable (46.9% and 46.8% in cross-sections I and II, respectively).

After age-standardization, these prevalence rates remained stable for cortical cataract (23.8% and 23.5% in the two surveys) and PSC (6.3% and 5.9%). The slightly increased prevalence of nuclear cataract (from 18.7% to 24.2%) was not altered.

Table 2 shows the age-specific prevalence rates for cortical cataract, PSC and nuclear cataract in cross-sections I and II. A similar trend of increasing cataract prevalence with increasing age was evident for all three types of cataract in both surveys. Comparing the age-specific prevalence between the two surveys, a reduction in PSC prevalence in cross-section II was observed in the older age groups (≥ 75 years). In contrast, increased nuclear cataract prevalence in cross-section II was observed in the older age groups (≥ 70 years). Age-specific cortical cataract prevalence was relatively consistent between the two surveys, except for a reduction in prevalence observed in the 80–84 age group and an increasing prevalence in the older age groups (≥ 85 years).

**Table 2:** Age-specific prevalence of cataract types in cross sections I and II.

Cataract type	Age (years)	Cross-section I		Cross-section II	
		
		n	% (95% CL)*	n	% (95% CL)*
Cortical	50–54	473	4.4 (2.6–6.3)	338	7.4 (4.6–10.2)
	55–59	522	9.2 (6.7–11.7)	542	9.0 (6.6–11.5)
	60–64	615	16.4 (13.5–19.4)	556	16.7 (13.6–19.8)
	65–69	653	26.2 (22.8–29.6)	581	23.6 (20.1–27.0)
	70–74	516	31.2 (27.2–35.2)	514	35.4 (31.3–39.6)
	75–79	366	40.2 (35.1–45.2)	332	39.8 (34.5–45.1)
	80–84	194	58.8 (51.8–65.8)	163	42.9 (35.3–50.6)
	85–89	74	52.7 (41.1–64.4)	73	54.8 (43.1–66.5)
	90+	22	68.2 (47.0–89.3)	14	78.6 (54.0–103.2)
					
PSC	50–54	474	2.7 (1.3–4.2)	338	2.4 (0.7–4.0)
	55–59	522	2.9 (1.4–4.3)	541	2.6 (1.3–3.9)
	60–64	616	4.6 (2.9–6.2)	548	5.7 (3.7–7.6)
	65–69	655	6.3 (4.4–8.1)	573	4.5 (2.8–6.3)
	70–74	517	6.8 (4.6–8.9)	505	9.7 (7.1–12.3)
	75–79	367	11.4 (8.2–14.7)	327	9.5 (6.3–12.7)
	80–84	196	12.2 (7.6–16.9)	155	10.3 (5.5–15.2)
	85–89	74	18.9 (9.8–28.1)	69	11.6 (3.9–19.4)
	90+	23	21.7 (3.5–40.0)	11	0.0
					
Nuclear	50–54	323	1.6 (0.2–2.9)	331	0.9 (–0.2–1.9)
	55–59	386	2.3 (0.8–3.8)	507	3.6 (1.9–5.2)
	60–64	453	5.3 (3.2–7.4)	501	11.6 (8.8–14.4)
	65–69	478	17.2 (13.8–20.1)	534	18.5 (15.2–21.9)
	70–74	392	27.6 (23.1–32.0)	453	36.0 (31.6–40.4)
	75–79	255	45.1 (39.0–51.3)	302	55.6 (50.0–61.3)
	80–84	146	54.1 (45.9–62.3)	147	73.5 (66.3–80.7)
	85–89	50	64.0 (50.2–77.8)	70	80.0 (70.4–89.6)
	90+	18	72.2 (49.3–95.1)	15	73.3 (48.0–98.7)

n = number of persons

* 95% Confidence Limits

Similar gender differences in cataract prevalence were observed in both surveys (Table 3). Higher prevalence of cortical and nuclear cataract in women than men was evident but the difference was only significant for cortical cataract (age-adjusted odds ratio, OR, for women 1.3, 95% confidence intervals, CI, 1.1–1.5 in cross-section I and OR 1.4, 95% CI 1.1–1.6 in cross-section II). In contrast, men had slightly higher PSC prevalence than women in both cross-sections but the difference was not significant (OR 1.1, 95% CI 0.8–1.4 for men in cross-section I and OR 1.2, 95% 0.9–1.6 in cross-section II).

**Table 3:** Gender distribution of cataract types in cross-sections I and II.

Cataract type	Gender	Cross-section I		Cross-section II	
		
		n	% (95% CL)*	n	% (95% CL)*
Cortical	Male	1496	21.1 (19.0–23.1)	1328	20.4 (18.2–22.6)
	Female	1939	25.9 (23.9–27.8)	1785	26.2 (24.2–28.3)
					
PSC	Male	1500	6.5 (5.2–7.7)	1314	6.4 (5.1–7.7)
	Female	1944	6.2 (5.1–7.2)	1753	5.7 (4.6–6.7)
					
Nuclear	Male	1106	17.6 (15.4–19.9)	1225	22.5 (20.1–24.8)
	Female	1395	19.5 (17.4–21.6)	1635	25.0 (22.9–27.1)

n = number of persons

* 95% Confidence Limits

## Discussion

Findings from two surveys of BMES cross-sectional populations with similar age and gender distribution showed that the prevalence of cortical cataract and PSC remained stable, while the prevalence of nuclear cataract appeared to have increased. Comparison of age-specific prevalence, with totally independent samples within each age group, confirmed the robustness of our findings from the two survey samples. Although lens photographs taken from the two surveys were graded for nuclear cataract by the same graders, who documented a high inter- and intra-grader reliability, we cannot exclude the possibility that variations in photography, performed by different photographers, may have contributed to the observed difference in nuclear cataract prevalence. However, the overall prevalence of any cataract (including cataract surgery) was relatively stable over the 6-year period.

Although different population-based studies used different grading systems to assess cataract [15], the overall prevalence of the three cataract types were similar across different study populations [12,16-23]. Most studies have suggested that nuclear cataract is the most prevalent type of cataract, followed by cortical cataract [16-20]. Ours and other studies reported that cortical cataract was the most prevalent type [12,21-23].

Our age-specific prevalence data show a reduction of 15.9% in cortical cataract prevalence for the 80–84 year age group, concordant with an increase in cataract surgery prevalence by 9% in those aged 80+ years observed in the same study population [10]. Although cortical cataract is thought to be the least likely cataract type leading to a cataract surgery, this may not be the case in all older persons.

A relatively stable cortical cataract and PSC prevalence over the 6-year period is expected. We cannot offer a definitive explanation for the increase in nuclear cataract prevalence. A possible explanation could be that a moderate level of nuclear cataract causes less visual disturbance than the other two types of cataract, thus for the oldest age groups, persons with nuclear cataract could have been less likely to have surgery unless it is very dense or co-existing with cortical cataract or PSC. Previous studies have shown that functional vision and reading performance were high in patients undergoing cataract surgery who had nuclear cataract only compared to those with mixed type of cataract (nuclear and cortical) or PSC [24,25]. In addition, the overall prevalence of any cataract (including cataract surgery) was similar in the two cross-sections, which appears to support our speculation that in the oldest age group, nuclear cataract may have been less likely to be operated than the other two types of cataract. This could have resulted in an increased nuclear cataract prevalence (due to less being operated), compensated by the decreased prevalence of cortical cataract and PSC (due to these being more likely to be operated), leading to stable overall prevalence of any cataract.

Possible selection bias arising from selective survival among persons without cataract could have led to underestimation of cataract prevalence in both surveys. We assume that such an underestimation occurred equally in both surveys, and thus should not have influenced our assessment of temporal changes.

Measurement error could also have partially contributed to the observed difference in nuclear cataract prevalence. Assessment of nuclear cataract from photographs is a potentially subjective process that can be influenced by variations in photography (light exposure, focus and the slit-lamp angle when the photograph was taken) and grading. Although we used the same Topcon slit-lamp camera and the same two graders who graded photos from both surveys, we are still not able to exclude the possibility of a partial influence from photographic variation on this result.

A similar gender difference (women having a higher rate than men) in cortical cataract prevalence was observed in both surveys. Our findings are in keeping with observations from the Beaver Dam Eye Study [18], the Barbados Eye Study [22] and the Lens Opacities Case-Control Group [26]. It has been suggested that the difference could be related to hormonal factors [18,22]. A previous study on biochemical factors and cataract showed that a lower level of iron was associated with an increased risk of cortical cataract [27]. No interaction between sex and biochemical factors were detected and no gender difference was assessed in this study [27]. The gender difference seen in cortical cataract could be related to relatively low iron levels and low hemoglobin concentration usually seen in women [28]. Diabetes is a known risk factor for cortical cataract but in this particular population diabetes is more prevalent in men than women in all age groups [29]. Differential exposures to cataract risk factors or different dietary or lifestyle patterns between men and women may also be related to these observations and warrant further study.

## Conclusion

In summary, in two population-based surveys 6 years apart, we have documented a relatively stable prevalence of cortical cataract and PSC over the period. The observed overall increased nuclear cataract prevalence by 5% over a 6-year period needs confirmation by future studies, and reasons for such an increase deserve further study.

## Competing interests

The author(s) declare that they have no competing interests.

## Authors' contributions

AGT graded the photographs, performed literature search and wrote the first draft of the manuscript. JJW graded the photographs, critically reviewed and modified the manuscript. ER performed the statistical analysis and critically reviewed the manuscript. PM designed and directed the study, adjudicated cataract cases and critically reviewed and modified the manuscript. All authors read and approved the final manuscript.

## Pre-publication history

The pre-publication history for this paper can be accessed here:


